# Horse Kick to the Abdomen Causing a Triad of Injury: A Case Report

**DOI:** 10.7759/cureus.5821

**Published:** 2019-10-01

**Authors:** Mohamed Ahmed, Rasha Saeed, May Abdulsalam, Samir Johna, Dina Elias

**Affiliations:** 1 Surgery, University of California, Riverside, USA; 2 Surgery, Arrowhead Regional Medical Center, Fontana, USA; 3 Family Practice, Ibn Albaldi Hospital, Baghdad, IRQ; 4 Surgery, Loma Linda University School of Medicine, Loma Linda, USA; 5 Trauma, Riverside Community Hospital, Riverside, USA

**Keywords:** blunt trauma, small bowel perforation, traumatic spine injury, rectus abdominis

## Abstract

A 35-year-old male, a horse trainer, was brought to the emergency room after being kicked in the abdomen, which resulted in an abdominal wall hematoma and a blow-out rupture of the proximal jejunum, with a mesenteric tear and posterior lumbar disc herniation. The initial evaluation did not raise significant concerns; however, the patient's abdominal pain progressively worsened after the administration of oral contrast in preparation for the computed tomography (CT) scan. The patient did well after abdominal exploration and operative repair of the small bowel injury. Our objective is to shed light on this mechanism of injury that can be underestimated during a patient's initial evaluation.

## Introduction

In the western world, the role of the horse has changed from that of a work animal to sports activities. More than 100,000 cases of horse-riding accidents are reported annually in the US, with long bone fractures and head injury being the most common [1-2]. Reported cases in the literature of trauma caused by a horse kick to the abdomen are scarce [3-4]. We report a triad of injuries caused by a horse kick to the abdomen.

## Case presentation

**Figure 1:**
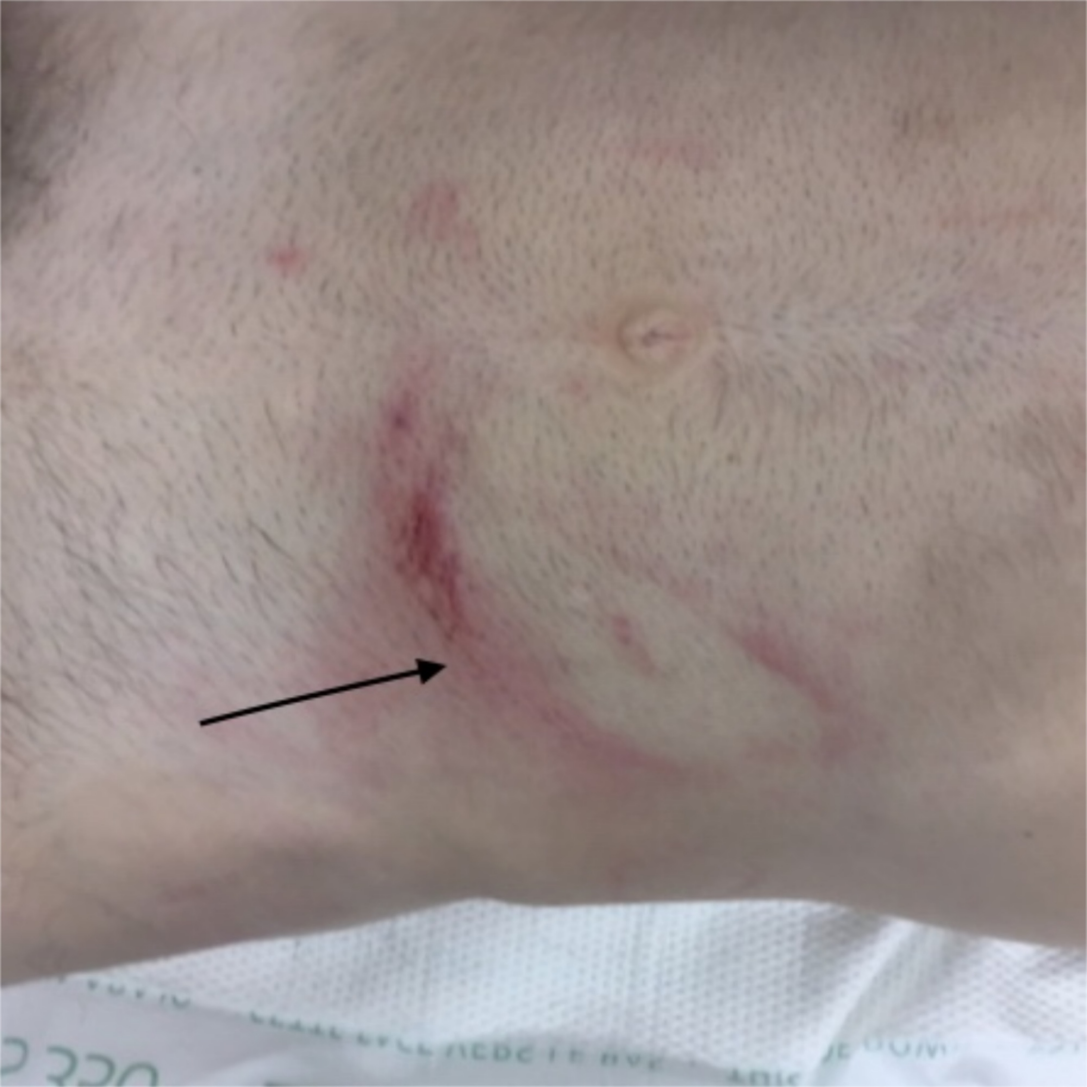
Abdominal wall bruising Horse hoof imprint on the left lower quadrant of the abdominal wall (black arrow)

A 35-year-old male horse trainer was brought to our emergency room after being kicked by a horse and complaining of left-sided abdominal pain, mainly at the hoof imprint site. The patient was hemodynamically stable, with a tender left lower abdomen at the site of the hoof mark (Figure 1 ). His pain worsened shortly after drinking oral contrast.

**Figure 2:**
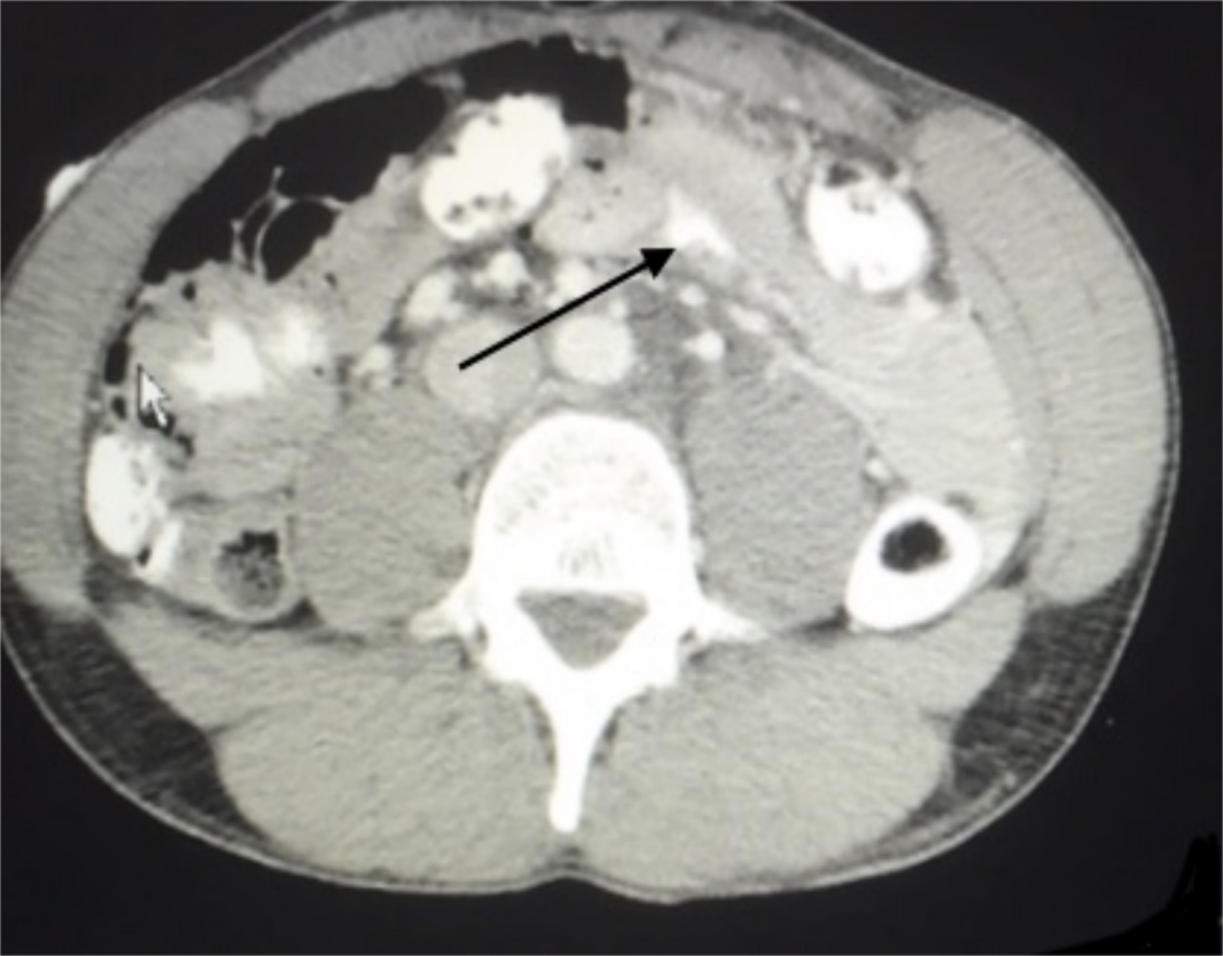
CT scan abdomen Oral contrast extravasation (black arrow) CT: computed tomography

On arrival, focused assessment with sonography for trauma was negative and a computed tomography (CT) scan of the abdomen and pelvis with oral, rectal, and intravenous contrast revealed extensive extravasation of bowel contrast (Figure 2) and posterior lumbar disc herniation.

**Figure 3:**
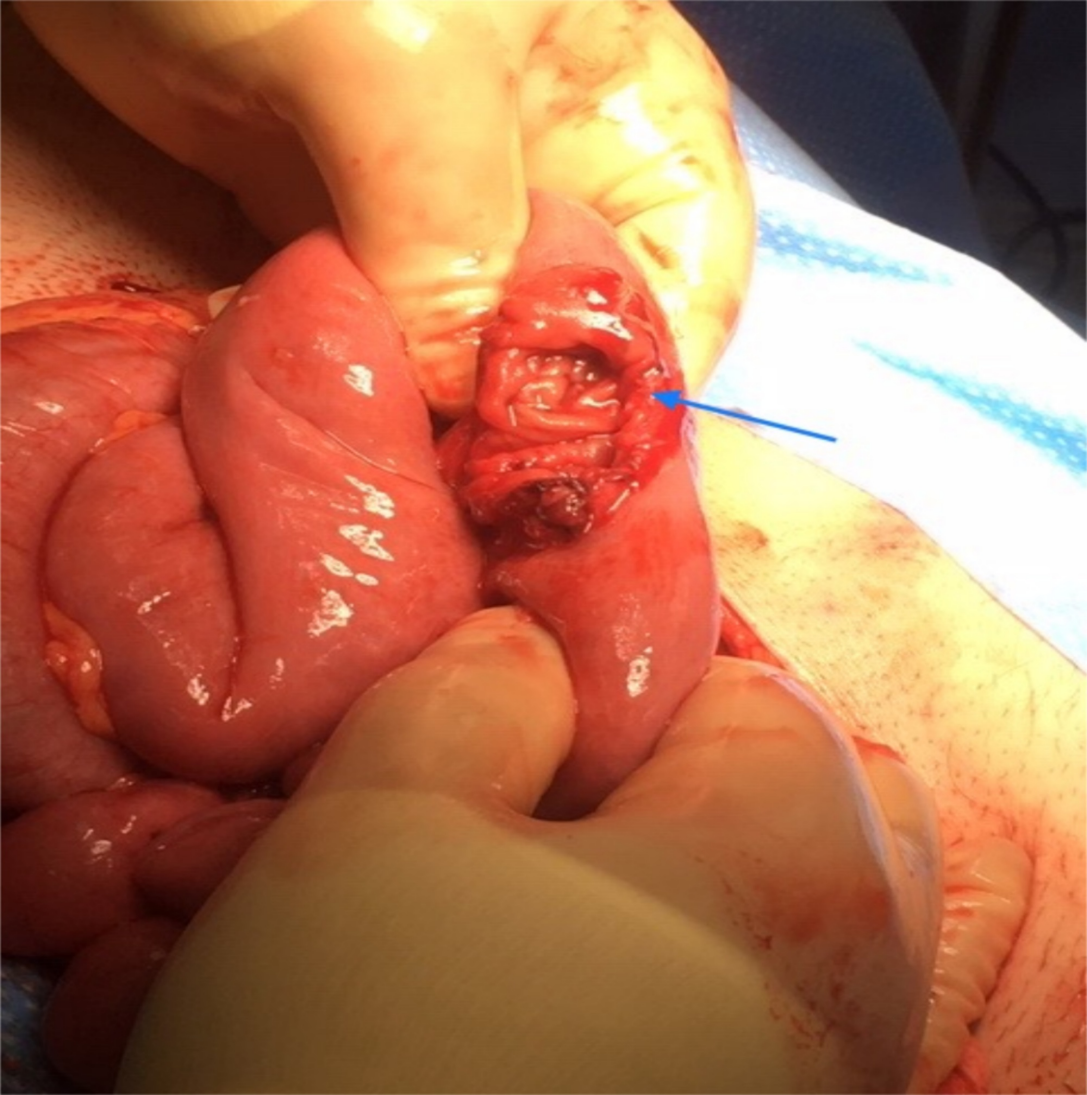
Operative finding Blow-out rupture of the small bowel (blue arrow)

The patient was taken to the operating room and an abdominal exploration was performed. A blow-out rupture of the proximal jejunum, a mesentery tear, and a stable rectus sheet hematoma were found (Figure 3).

**Figure 4:**
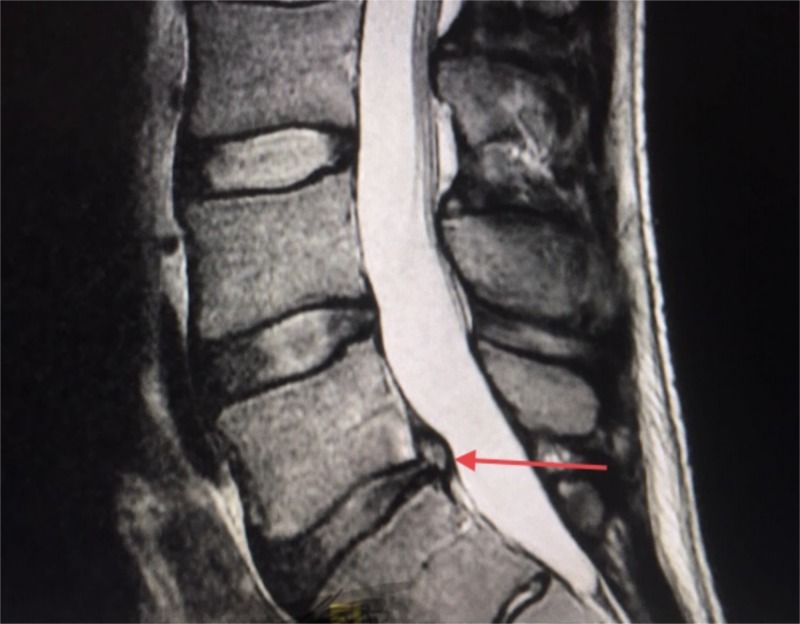
Magnetic resonance imaging of the lumbar spine Disc posterior herniation (red arrow)

The small bowel perforation was repaired and wash-out performed. Magnetic resonance imaging (MRI) of the lumbar spine revealed multilevel disc protrusions most significant at L4-5 (Figure 4). No operative management was recommended by neurosurgery.

The patient did well and was discharged from the hospital on the third postoperative day.

## Discussion

The ancient Arabic proverb "The grave yawns for the horseman" describes the possible lethal power of a horse, which is capable of delivering a kick with a force of up to one ton [5]. The mechanism of injury includes a fall from a running horse, a kick, a bite, or being dragged [6]. In the largest series reported by Carmichael et al., most injuries occurred due to falling off while riding (54%) or sustaining a kick from the horse (22%), resulting in extremity fractures (33%) and head injury (27%) [7]. Extremity injuries are the most common horseback riding-related injuries (upper more than lower) and the head is the most frequent site of injury when kicked by a horse [8-9]. Cardiac injury in the form of myocardial infarction [10], traumatic tricuspid regurgitation, right-to-left intra-atrial shunt, persistent complete heart block [11], and cardiac tamponade following a horse kick have been reported [12]. Injuries are usually recognized early, however, delayed diagnoses of cardiac tamponade and retroperitoneal rupture of the duodenum have also been reported [13-14]. The transfer of energy from the end of the hoof to a small field leads to internal injuries that are more severe than predicted, similar to a handlebar injury [15].

## Conclusions

A horse kick delivers significant force to a small surface area, which should never be underestimated even in patients whose initial exam raises no concerns. Internal organ injuries may not be apparent at initial evaluation and early CT imaging is advised. Other associated injuries should not be overlooked and, in our case, a posterior disc herniation was clearly defined with the use of MRI.

## References

[1] McCrory P, Turner M (2005). Equestrian injuries. Med Sport Sci.

[2] Eckert V, Lockemann U, Püschel K, Meenen N, Hessler C (2011). Equestrian injuries caused by horse kicks: first results of a prospective multicenter study. Clin J Sport Med.

[3] Nogalski A, Jankiewicz L, Cwik G, Karski J, Matuszewski Ł (2007). Animal related injuries treated at the Department of Trauma and Emergency Medicine, Medical University of Lublin. Ann Agric Environ Med.

[4] Yim W, Yeung H, Mak S, Graham CA, Lai PBS, Rainer TH (2007). Five year analysis of Jockey Club horse-related injuries presenting to a trauma centre in Hong Kong. Injury.

[5] Pounder DJ (1984). "The grave yawns for the horseman." Equestrian deaths in South Australia 1973-1983. Med J Aust.

[6] Newton AM, Nielsen AM (2005). A review of horse-related injuries in a rural Colorado hospital: implications for outreach education. J Emerg Nurs.

[7] Carmichael SP, Davenport DL, Kearney PA, Bernard AC (2014). On and off the horse: mechanisms and patterns of injury in mounted and unmounted equestrians. Injury.

[8] Bixby-Hammett D, Brooks WH (1990). Common injuries in horseback riding. Sports Med.

[9] Exadaktylos A, Eggli S, Inden P, Zimmermann H (2002). Hoof kick injuries in unmounted equestrians. Improving accident analysis and prevention by introducing an accident and emergency based relational database. Emerg Med J.

[10] Pérez A, Marcuschamer J, Sánchez G (1986). Myocardial infarct secondary to a non-penetrating chest injury. Presentation of a case and review of the literature [Article in Spanish]. Arch Inst Cardiol Mex.

[11] Benitez RM, Gold MR (1999). Immediate and persistent complete heart block following a horse kick. Pacing Clin Electrophysiol.

[12] Byrne R, Fleming S, Tolan M, Brown A (2010). Traumatic tricuspid regurgitation and right-to-left intra-atrial shunt--an unusual complication of a horse-kick. Ir Med J.

[13] Thomas TPE, Hinshaw AH (1956). Retroperitoneal rupture of the duodenum caused by blunt trauma with a case report. Ann Surg.

[14] Dunsire M, Field J, Valentine S (2001). Delayed diagnosis of cardiac tamponade following isolated blunt abdominal trauma. Br J Anaesth.

[15] Cherniawsky H, Bratu I, Rankin T, Sevcik W (2014). Serious impact of handlebar injuries. Clin Pediatr.

